# Impact of AMP-Activated Protein Kinase α1 Deficiency on Tissue Injury following Unilateral Ureteral Obstruction

**DOI:** 10.1371/journal.pone.0135235

**Published:** 2015-08-18

**Authors:** Sobuj Mia, Giuseppina Federico, Martina Feger, Tatsiana Pakladok, Adrian Meissner, Jakob Voelkl, Hermann-Josef Groene, Ioana Alesutan, Florian Lang

**Affiliations:** 1 Department of Physiology, University of Tübingen, Gmelinstr. 5, D-72076, Tübingen, Germany; 2 Department of Cellular and Molecular Pathology, German Cancer Research Center, Im Neuenheimer Feld, 280, 69120, Heidelberg, Germany; Université catholique de Louvain, BELGIUM

## Abstract

**Background:**

AMP-activated protein kinase (Ampk) is a sensor of the cellular energy status and a powerful regulator of metabolism. Activation of Ampk was previously shown to participate in monocyte-to-fibroblast transition and matrix protein production in renal tissue. Thus, the present study explored whether the catalytic Ampkα1 isoform participates in the regulation of the renal fibrotic response following unilateral ureteral obstruction (UUO).

**Methods:**

UUO was induced in gene-targeted mice lacking functional Ampkα1 (Ampkα1^-/-^) and in corresponding wild-type mice (Ampkα1^+/+^). In the obstructed kidney and, for comparison, in the non-obstructed control kidney, quantitative RT-PCR, Western blotting and immunostaining were employed to determine transcript levels and protein abundance, respectively.

**Results:**

In Ampkα1^+/+^ mice, UUO significantly up-regulated the protein abundance of the Ampkα1 isoform, but significantly down-regulated the Ampkα2 isoform in renal tissue. Phosphorylated Ampkα protein levels were significantly increased in obstructed kidney tissue of Ampkα1^+/+^ mice but not of Ampkα1^-/-^ mice. Renal expression of α-smooth muscle actin was increased following UUO, an effect again less pronounced in Ampkα1^-/-^ mice than in Ampkα1^+/+^ mice. Histological analysis did not reveal a profound effect of Ampkα1 deficiency on collagen 1 protein deposition. UUO significantly increased phosphorylated and total Tgf-ß-activated kinase 1 (Tak1) protein, as well as transcript levels of Tak1-downstream targets *c-Fos*, *Il6*, *Pai1* and *Snai1* in Ampkα1^+/+^ mice, effects again significantly ameliorated in Ampkα1^-/-^ mice. Moreover, Ampkα1 deficiency inhibited the UUO-induced mRNA expression of *Cd206*, a marker of M2 macrophages and of *Cxcl16*, a pro-fibrotic chemokine associated with myeloid fibroblast formation. The effects of Ampkα1 deficiency during UUO were, however, paralleled by increased tubular injury and apoptosis.

**Conclusions:**

Renal obstruction induces an isoform shift from Ampkα2 towards Ampkα1, which contributes to the signaling involved in cell survival and fibrosis.

## Introduction

Renal fibrosis, characterized by excessive accumulation of extracellular matrix proteins, is a hallmark of several renal diseases [1, 2]. Renal fibrosis is considered as inappropriate wound healing that occurs after chronic and sustained injury [3]. Progressive renal fibrosis leads ultimately to end-stage renal failure [3]. Extracellular matrix proteins are accumulated by myofibroblasts [4], which could originate from various sources [4–6]. Myofibroblasts are recognized by expression of mesenchymal proteins including α-smooth muscle actin (α-Sma) and collagen [7].

Signaling leading to the renal fibrotic response [8] include Tgf-β [9], which exerts its effects via Smad and non-Smad signaling pathways [10, 11]. Tgf-β-activated kinase 1 (Tak1) is one of the major kinases through which Tgf-β mediates its pro-fibrotic actions independently of Smad [12]. Therefore, Tak1 has been considered as a converging point of pro-fibrotic signaling in renal fibrosis [12]. Tak1 has been shown to activate the AMP-activated protein kinase (Ampk) [13]. On the other hand, Ampk has been shown to activate Tak1 and mediate pro-inflammatory effects [14]. Tak1-sensitive signaling includes transcription factors *c-Fos* [15] and *Snai1* [15], as well as the inflammatory mediators interleukin *Il6* [16] and plasminogen activator inhibitor *Pai1* [15].

Ampk is composed of a catalytic α subunit and regulatory β and γ subunits [17]. Two catalytic α subunit isoforms have been identified, i.e. the ubiquitously expressed Ampkα1 prevailing in the non-nuclear fraction and the mainly in skeletal muscle and heart expressed Ampkα2, localized in both the nucleus and the non-nuclear fraction [17, 18]. The two catalytic Ampkα isoforms differ in targets and effects [19, 20].

Ampk is a sensor of the cytosolic AMP/ATP concentration ratio and is activated following cellular energy depletion [21, 22]. The kinase is further stimulated by a decrease of O_2_ levels [23] and an increase of cytosolic Ca^2+^ activity [21, 24, 25]. Ampk stimulates a variety of cellular functions in order to up-regulate cellular ATP production [26] including cellular glucose uptake, glycolysis and fatty acid oxidation [22, 27, 28]. Moreover, Ampk curtails energy consumption by inhibiting ATP-dependent processes including protein synthesis, gluconeogenesis and lipogenesis [22, 26, 27]. Besides its role in cellular metabolism, Ampk regulates a variety of other signaling mechanisms [29]. Ampk contributes to the protection of cells during energy depletion [26, 30, 31]. Energy consumption in ischemic tissues could be decreased by replacement of oxygen consuming cells with fibrous tissue [32]. On the other hand, Ampk has been shown to decrease fibrosis in fibroblasts and tubular cells [33], as well as fibrosis following unilateral ureteral obstruction (UUO) [34]. Moreover, Ampk activators have been shown to counteract Tgf-β-induced collagen stimulation [35].

The present study explored the impact of Ampkα1 isoform on myofibroblast formation and renal fibrosis following obstructive nephropathy [36, 37]. UUO mimics obstructive nephropathy and triggers renal extracellular matrix deposition and fibrosis [1]. UUO was induced in gene-targeted mice lacking functional Ampkα1 (Ampkα1^-/-^) and corresponding wild-type mice (Ampkα1^+/+^).

## Methods

### Ethics statement

All animal experiments were conducted according to the recommendations of the Guide for Care and Use of Laboratory Animals of the National Institutes of Health as well as the German law for welfare of animals, and reviewed and approved by the respective government authority of the state Baden-Württemberg (Regierungspräsidium) prior to the start of the study. All efforts were made to minimize animal suffering. Experiments have been performed in gene-targeted mice completely lacking functional Ampkα1 (Ampkα1^-/-^) and in corresponding wild-type mice (Ampkα1^+/+^) [38].

### Unilateral ureteral obstruction

Renal fibrosis was induced by unilateral ureteral obstruction (UUO) [1, 39, 40]. Following surgical incision, the left ureter was exposed and ligated twice with a non-resorbable 7–0 filament. Following ligation the surgical wound was closed by sutures. Mice were treated with metamizole for analgesia (200 mg/kg BW) after the procedure and for the duration of the UUO experiment in drinking water. No blinding was applied. Mice were sacrificed by exsanguination and cervical dislocation under isoflurane anaesthesia. The mice were sacrificed 3 days, 7 days or 3 weeks after the ligation procedure and the obstructed as well as the non-ligated kidney rapidly removed and kidney tissues snap frozen in liquid nitrogen or fixed in 4% PFA. For the blood count, blood was analyzed 7 days after unilateral ureteral obstruction using a pocH-100iv automatic hematology analyzer (Sysmex).

### Quantitative RT-PCR

Total RNA was isolated from murine kidney tissues using Trifast Reagent (Peqlab) according to the manufacturer’s instructions. Reverse transcription of 2 μg RNA was performed using oligo(dT)_12–18_ primers (Invitrogen) and SuperScript III Reverse Transcriptase (Invitrogen). Quantitative real-time PCR was performed with the iCycler iQ^TM^ Real-Time PCR Detection System (Bio-Rad Laboratories) and iQ Sybr Green Supermix (Bio-Rad Laboratories) according to the manufacturer’s instructions. The following primers were used (5’→3’ orientation):

*a-Sma* fw: CCCAGACATCAGGGAGTAATGG;

*a-Sma* rev: CTATCGGATACTTCAGCGTCA;

*Bax* fw: AGACAGGGGCCTTTTTGCTAC;

*Bax* rev: AATTCGCCGGAGACACTCG;

*Bcl2* fw: ATGCCTTTGTGGAACTATATGGC;

*Bcl2* rev: GGTATGCACCCAGAGTGATGC;

*Cd206* fw: GAGGGAAGCGAGAGATTATGGA;

*Cd206* rev: GCCTGATGCCAGGTTAAAGCA;

*c-Fos* fw: CGGCATCATCTAGGCCCAG;

*c-Fos* rev: TCTGCTGCATAGAAGGAACCG;

*Col1a1*fw:ACCCGAGGTATGCTTGATCTG;

*Col1a1*rev:CATTGCACGTCATCGCACAC;

*Col3a1*fw:CCATTTGGAGAATGTTGTGCAAT;

*Col3a1*rev:GGACATGATTCACAGATTCCAGG;

*Cxcl16* fw: ATACCGCAGGGTACTTTGGAT;

*Cxcl16* rev: CTGCAACTGGAACCTGATAAAGA;

*Il6* fw: TCTATACCACTTCACAAGTCGGA;

*Il6* rev: GAATTGCCATTGCACAACTCTTT;

*Gapdh* fw: AGGTCGGTGTGAACGGATTTG;

*Gapdh* rev: TGTAGACCATGTAGTTGAGGTCA;

*Pai-1* fw: TTCAGCCCTTGCTTGCCTC;

*Pai-1* rev: ACACTTTTACTCCGAAGTCGGT;

*Snai1* fw: CACACGCTGCCTTGTGTCT;

*Snai1* rev: GGTCAGCAAAAGCACGGTT.

The specificity of the PCR products was confirmed by analysis of the melting curves. All PCRs were performed in duplicate, and mRNA fold changes were calculated by the 2^-ΔΔCt^ method using Gapdh as internal reference. Results are shown normalized to the mRNA expression in the obstructed kidney tissues of wild-type mice (arbitrary units, a.u.).

### Western blot analysis

Murine kidney tissues were lysed with ice-cold lysis buffer (Thermo Fisher Scientific) supplemented with complete protease and phosphatase inhibitor cocktail (Thermo Fisher Scientific). After centrifugation at 10000 rpm for 5 min, proteins were boiled in Roti-Load1 Buffer (Carl Roth, Karlsruhe, Germany) at 100°C for 5 min. Proteins were separated on SDS-polyacrylamide gels and transferred to PVDF membranes. The membranes were incubated overnight at 4°C with the following primary antibodies: rabbit anti-α-smooth muscle actin, rabbit anti-collagen I (diluted 1:1000, Abcam), rabbit anti-phospho-AMPKα Thr^172^, rabbit anti-AMPKα, rabbit anti-phospho-ACC (Ser^79^), rabbit anti-ACC, rabbit anti-phospho-TAK1 Ser^412^, rabbit anti-TAK1, rabbit anti-phospho-Smad2 (Ser^465/467^), rabbit anti-Smad2, rabbit anti-GAPDH, rabbit anti-Tgf-β (diluted 1:1000, Cell Signaling), goat anti-Ampka2 (used at a 1:2000 dilution, Santa Cruz), rabbit anti-AMPKα1 (diluted 1:1000, Novus Biologicals) and then with secondary goat anti-rabbit HRP-conjugated antibody (diluted 1:1000, Cell Signaling) or donkey anti-goat HRP-conjugated antibody (diluted 1:1000, Santa Cruz) for 1 hour at room temperature. For loading controls, the membranes were stripped with stripping buffer (Carl Roth) at 60°C for 5 min. Antibody binding was detected with the ECL detection reagent (Thermo Fisher Scientific). Bands were quantified with Quantity One Software (Bio-Rad Laboratories) and results are shown normalized to the protein expression in the obstructed kidney tissues of wild-type mice (arbitrary units, a.u.).

### Histology and immunostaining

Immunostaining was performed on 3 μm sections of paraffin-embedded kidney tissue. The following primary antibodies have been used: mouse monoclonal α-smooth muscle actin (α-SMA) (1:200, Sigma), and rabbit CollagenI/III (1:20, Biotrend), followed by biotinylated anti-mouse Ig secondary antibody (1:200, Dianova) and biotinylated anti-rabbit secondary antibody (1:150, Dianova), respectively. Control sections were stained without primary antibody. As substrate for the reaction AEC kit (BioGenex) was applied. The CollagenI/III staining has been evaluated as 0- no staining, detectable, 1- mild, 2-moderate, and 3- intense staining. A degree-specific staining index has been defined as the percentage of fields with the respective degree of injury, and the total staining score index has been calculated as the sum of specific damage indices, whereby the index with degree 1 was multiplied by 1, that of degree 2 by 2, that of degree 3 by 3. Morphometric analysis has been performed on PAS staining using a semiautomatic image analyzing system (Leica Q600 Qwin; Leica Microsystems), and the percentage of differentiated proximal tubules has been evaluated by examining at least 15 fields of cortex and inner medulla at a magnification of 20X, obtained after exclusion of glomeruli. *In Situ* Cell Death Detection kit, POD (Tunel technology) (Roche) has been used to visualize apoptotic cells. The number of Tunel-positive cells has been counted in at least 10 fields at a magnification of 40X.

### Statistics

Data are provided as means ± SEM, *n* represents the number of independent experiments. Data were tested for significance between genotypes using Student t-test (normal distributed data) or Mann-Whitney test (non-normal distributed data) according to Shapiro-Wilk test. Data were tested between non-obstructed control kidneys and obstructed kidneys for significance by paired t-test or Wilcoxon test. Only results with *p*<0.05 were considered statistically significant. If multiple comparisons were made, Bonferoni correction was applied.

## Results

The present study explored the role of Ampkα1 isoform in the development of renal tissue fibrosis following obstructive nephropathy. To this end, gene-targeted mice lacking functional Ampkα1 (Ampkα1^-/-^) and corresponding wild-type mice (Ampkα1^+/+^) were subjected to unilateral ureteral obstruction (UUO). Seven days later, the animals were sacrificed and the obstructed kidney was compared to the non-obstructed control kidney. In agreement with earlier observations [30], red blood cell count was decreased and spleen weight increased in Ampkα1^-/-^ mice as compared to Ampkα1^+/+^ mice (Table 1).

**Table 1:** Anemia in Ampkα1-deficient mice.

	Ampkα1^+/+^	Ampkα1^-/-^
RBC* 10^6^ /μl	8.80 ± 0.20	6.93 ± 0.21 ***
WBC *10^3^ /μl	11.71 ± 1.59	9.65 ± 1.17
HGB [g/dl]	15.30 ± 0.26	11.30 ± 0.26 ***
HCT [%]	49.07 ± 1.20	37.70 ± 0.89 ***
MCV [fL]	55.76 ± 0.25	54.52 ± 0.65
MCH [pg]	17.39 ± 0.19	16.33 ± 0.16 **
MCHC [g/dl]	31.21 ± 0.36	29.98 ± 0.17 *
PLT*10^3^ /μl	1038.9 ± 38.3	1090.7 ± 74.0
Spleen w./ bw. [mg/g]	5.27 ± 0.44	11.99 ± 0.81 **

Arithmetic means ± SEM (n = 6–7) of blood parameters (red blood cell count (RBC), white blood cell count (WBC), hemoglobin concentration (HGB), hematocrit (HCT), mean corpuscular volume, (MCV), mean corpuscular hemoglobin (MCH), mean corpuscular hemoglobin concentration (MCHC) and platelet count (PLT)) and spleen weight to body weight ratio of Ampkα1 knockout mice (Ampkα1^-/-^) and respective wild-type mice (Ampkα1^+/+^) following 7 days of unilateral ureteral obstruction.

*(p<0.05), **(p<0.01), ***(p<0.001) statistically significant vs. respective wild-type mice.

**Fig 1:**
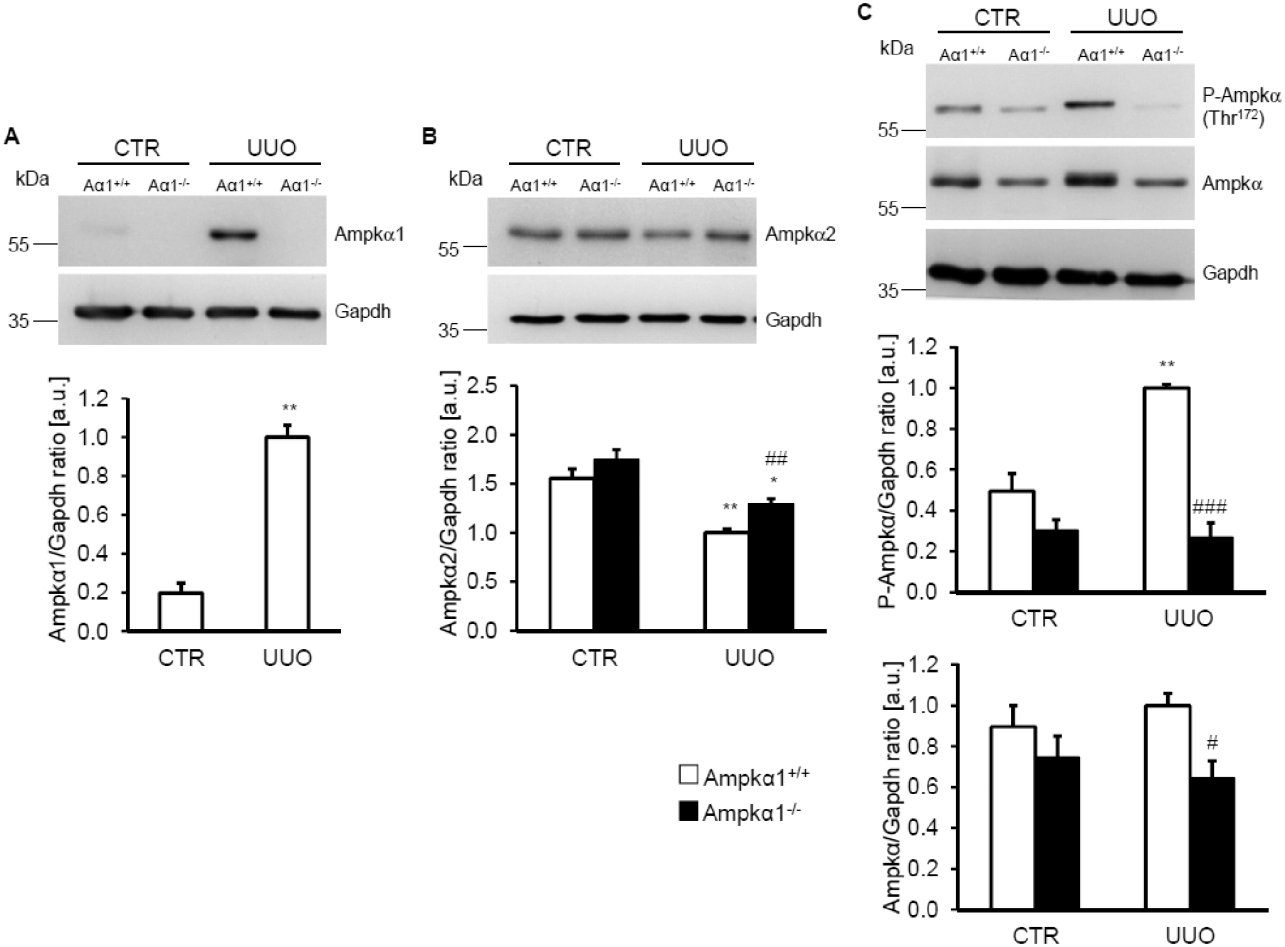
Renal Ampkα1 protein is induced following unilateral ureteral obstruction. **A.** Representative original Western blots showing Ampkα1 and Gapdh protein expression in renal tissue from non-obstructed control kidney (CTR) and obstructed kidney (UUO) of Ampkα1 knockout mice (Aα1^-/-^) and respective wild-type mice (Aα1^+/+^) following 7 days of unilateral ureteral obstruction. Arithmetic means ± SEM (n = 9) of normalized Ampkα1/Gapdh protein ratio in renal tissue from non-obstructed control kidney (CTR) and obstructed kidney (UUO) of wild-type mice (Ampkα1^+/+^) following 7 days of unilateral ureteral obstruction (UUO). **B.** Representative original Western blots and arithmetic means ± SEM (n = 9) of normalized Ampkα2/Gapdh protein ratio in renal tissue from non-obstructed control kidney (CTR) and obstructed kidney (UUO) of Ampkα1 knockout mice (black bars, Ampkα1^-/-^) and respective wild-type mice (white bars, Ampkα1^+/+^) following 7 days of unilateral ureteral obstruction. **C.** Representative original Western blots and arithmetic means ± SEM (n = 9) of normalized phospho-Ampkα (Thr^172^)/Gapdh and total Ampkα/Gapdh protein ratio in renal tissue from non-obstructed control kidney (CTR) and obstructed kidney (UUO) of Ampkα1 knockout mice (black bars, Ampkα1^-/-^) and respective wild-type mice (white bars, Ampkα1^+/+^) following 7 days of unilateral ureteral obstruction. *(p<0.05), **(p<0.01) statistically significant vs. control kidney tissues of respective mice; #(p<0.05), ##(p<0.01), ###(p<0.001) statistically significant vs. respective kidney tissues of wild-type mice.

As illustrated in Fig 1A, Ampkα1 protein was expressed in renal tissue of Ampkα1^+/+^ mice but not of Ampkα1^-/-^ mice. Ampkα1 protein expression was significantly up-regulated in obstructed kidney tissues of Ampkα1^+/+^ mice following 7 days of UUO. Similar effects on Ampkα1 protein expression were observed at earlier stages (Fig A in S2 File) and later stages (Fig A in S4 File) of UUO. In contrast, Ampkα2 isoform protein expression was not significantly modified by obstructive injury after 3 days of UUO (Fig B in S2 File), but significantly down-regulated in the obstructed kidney tissues as compared to non-obstructed kidney tissue of both Ampkα1^+/+^ mice and Ampkα1^-/-^ mice following 7 days (Fig 1B) and 3 weeks (Fig B in S4 File) of UUO. Ampkα2 isoform expression tended to be higher in the Ampkα1^-/-^ mice than in Ampkα1^+/+^ mice in both, the non-obstructed control kidney tissues and the obstructed kidney tissues (Fig 1B, Fig B in S2 File and Fig B in S4 File). Thus, UUO leads to an isoform shift towards Ampkα1 in renal tissue.

The abundance of phosphorylated Ampkα was low in the non-obstructed kidney tissue from both Ampkα1^-/-^ mice and Ampkα1^+/+^ mice. 3 days (Fig C in S2 File) and 7 days (Fig 1C) of UUO was followed by a significant increase of phosphorylated Ampkα protein in the obstructed kidney tissue as compared to the non-obstructed kidney tissue of Ampkα1^+/+^ mice but not of Ampkα1^-/-^ mice. Accordingly, the phosphorylation of Ampkα was significantly lower in the obstructed kidney tissue of Ampkα1^-/-^ mice than in the obstructed kidney tissues of Ampkα1^+/+^ mice. 3 weeks after UUO, phosphorylation of Ampkα tended to be lower in the obstructed kidney tissue than in the non-obstructed kidney tissue of Ampkα1^+/+^ mice, effects, however, not reaching statistical significance (Fig C in S4 File), but was virtually absent in the Ampkα1^-/-^ mice. Total Ampkα protein abundance was not significantly modified in the obstructed kidney tissue than in the non-obstructed kidney tissue of Ampkα1^+/+^ mice, but was significantly decreased in the obstructed kidney tissue of Ampkα1^-/-^ mice as compared to Ampkα1^+/+^ mice. To further elucidate whether UUO up-regulates Ampkα activity, the phosphorylation of the Ampk target protein acetyl-CoA carboxylase (Acc) [41] was quantified. After 7 days of UUO, the abundance of phosphorylated Acc and total Acc protein tended to be lower in Ampkα1^-/-^ kidney tissues than in Ampkα1^+/+^ kidney tissues, a difference, however, not reaching statistical significance (Fig A in S1 File).

**Fig 2:**
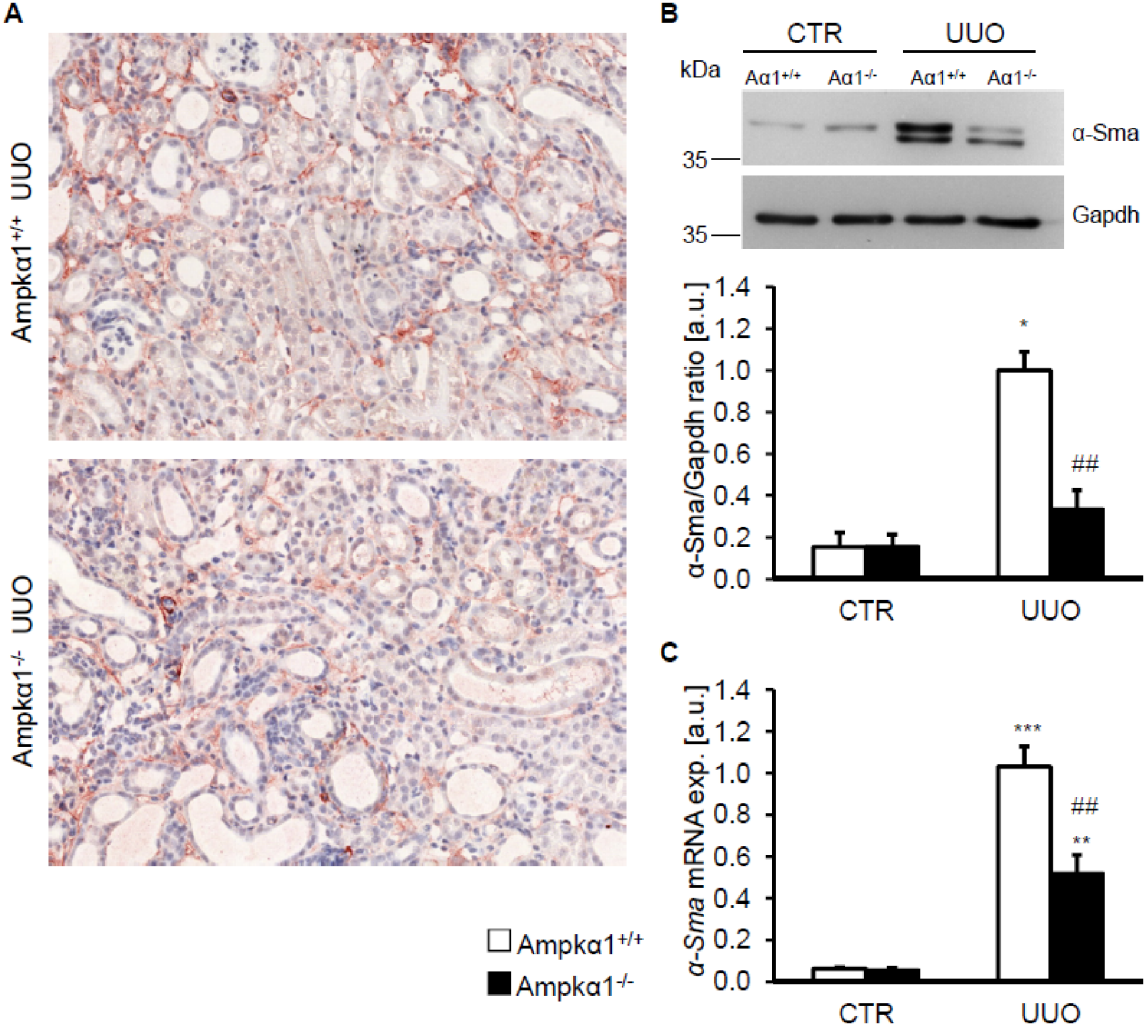
Ampkα1 sensitive up-regulation of α-smooth muscle actin following unilateral ureteral obstruction. **A.** Representative original histological images showing α-smooth muscle actin protein abundance in sections of obstructed kidneys (UUO) from Ampkα1 knockout mice (Ampkα1^-/-^) and respective wild-type mice (Ampkα1^+/+^) following 7 days of unilateral ureteral obstruction (magnification 200x). **B.** Representative original Western blots and arithmetic means ± SEM (n = 9) of normalized α-smooth muscle actin (α-Sma)/Gapdh protein ratio in renal tissue from non-obstructed control kidney (CTR) and obstructed kidney (UUO) of Ampkα1 knockout mice (black bars, Ampkα1^-/-^) and respective wild-type mice (white bars, Ampkα1^+/+^) following 7 days of unilateral ureteral obstruction. **C.** Arithmetic means ± SEM (n = 9) of *α-Sma* relative mRNA expression in renal tissue from non-obstructed control kidney (CTR) and obstructed kidney (UUO) of Ampkα1 knockout mice (black bars) and respective wild-type mice (white bars) following 7 days of unilateral ureteral obstruction. *(p<0.05), **(p<0.01), ***(p<0.001) statistically significant vs. control kidney tissues of respective mice; ##(p<0.01) statistically significant vs. respective kidney tissues of wild-type mice.

In order to test whether Ampkα1 modulates the myofibroblast formation in obstructive nephropathy, the expression of α-smooth muscle actin (α-Sma) was determined following 7 days of UUO. As shown in Fig 2A, the expression of α-Sma in obstructed kidney tissues was lower in Ampkα1^-/-^ mice than in Ampkα1^+/+^ mice. Accordingly, as determined by Western blotting and quantitative RT-PCR, the mRNA levels and protein abundance of α-Sma were significantly upregulated in obstructed kidney tissue as compared to non-obstructed kidney tissue of Ampkα1^+/+^ mice, effects significantly blunted by Ampkα1^-/-^ deficiency (Fig 2B and 2C). No significant effects of Ampkα1 deficiency on α-Sma expression were observed in early stage after 3 days of UUO (Fig A in S3 File). In contrast, at 3 weeks after UUO the increase in α-Sma expression in obstructed kidney tissue was again significantly attenuated of Ampkα1^-/-^ mice (Fig A in S5 File), suggesting that Ampkα1 modulates the response to obstructed injury rather at middle and late stages.

**Fig 3:**
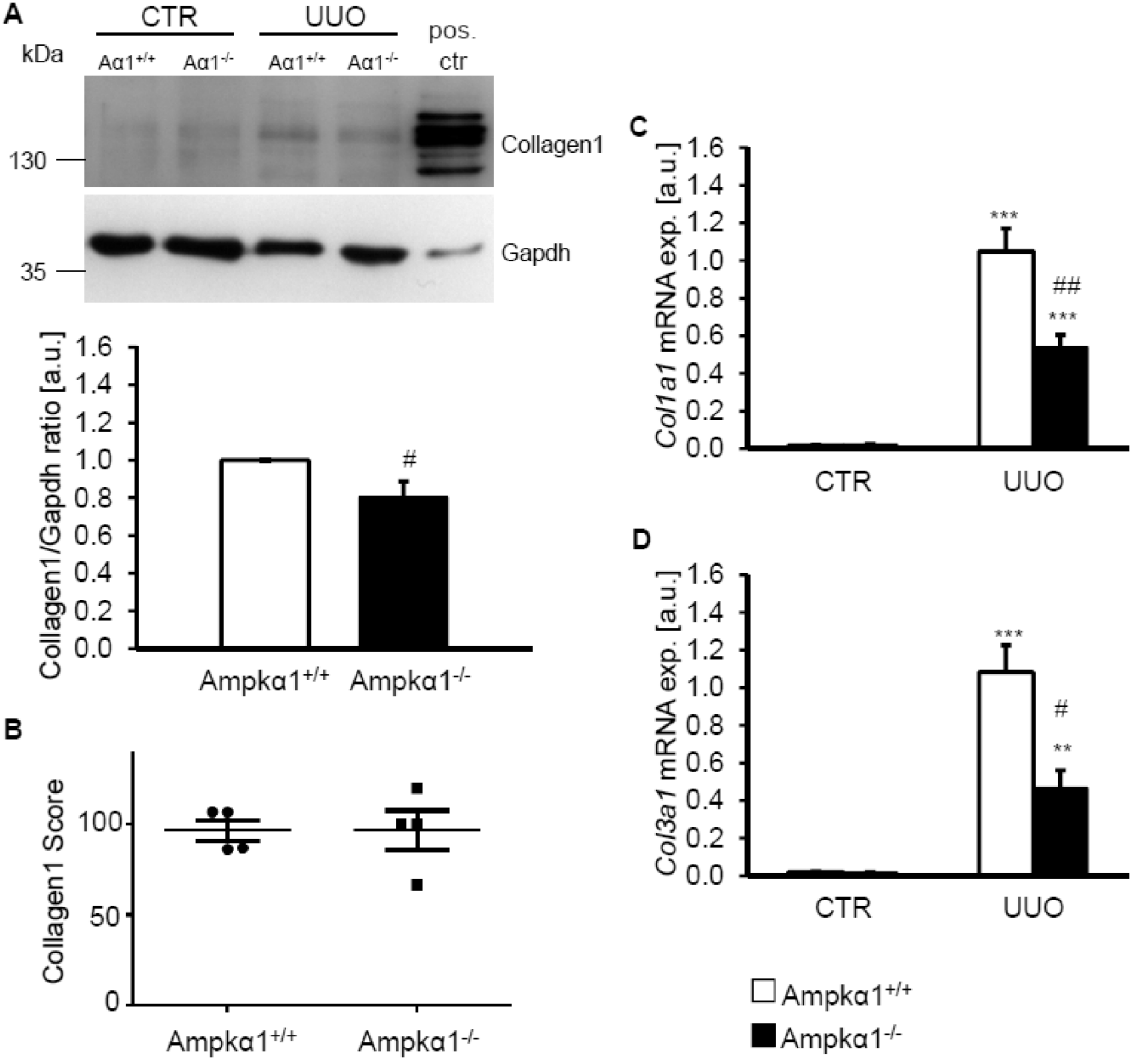
Ampkα1 deficiency attenuates collagen expression following unilateral ureteral obstruction. **A.** Representative original Western blots showing collagen type I and Gapdh protein expression in renal tissue from non-obstructed control kidney (CTR) and obstructed kidney (UUO) of Ampkα1 knockout mice (Aα1^-/-^) and respective wild-type mice (Aα1^+/+^) following 7 days of unilateral ureteral obstruction. Murine tendon tissues were used as positive control. Arithmetic means ± SEM (n = 9) of normalized Collagen type 1/Gapdh protein ratio in renal tissue from obstructed kidney of Ampkα1 knockout mice (black bar, Ampkα1^-/-^) and respective wild-type mice (white bar, Ampkα1^+/+^) following 7 days of unilateral ureteral obstruction. **B.** Semi-qualitative evaluation of Collagen type I/III deposition in sections of obstructed kidneys from Ampkα1 knockout mice (Ampkα1^-/-^) and respective wild-type mice (Ampkα1^+/+^) following 7 days of unilateral ureteral obstruction. Arithmetic means ± SEM (n = 9) of collagen type I (*Col1a1*, **C**) and collagen type III (*Col3a1*, **D**) relative mRNA expression in renal tissue from non-obstructed control kidney (CTR) and obstructed kidney (UUO) of Ampkα1 knockout mice (black bars) and respective wild-type mice (white bars) following 7 days of unilateral ureteral obstruction. **(p<0.01), ***(p<0.001) statistically significant vs. control kidney tissues of respective mice; #(p<0.05) ##(p<0.01) statistically significant vs. respective kidney tissues of wild-type mice.

Further experiments explored whether Ampkα1 modifies collagen synthesis. Following 7 days of UUO, the abundance of collagen type I protein was slightly, but significantly, lower in obstructed kidney tissues from Ampkα1^-/-^ mice than in obstructed kidney tissues from Ampkα1^+/+^ mice (Fig 3A). However, no difference in collagen type I deposition was observed in histological sections of obstructed kidney tissues between the genotypes (Fig 3B). Nonetheless, the mRNA expression of *Col1a1* and *Col3a1* was significantly higher in obstructed kidney tissue than in non-obstructed kidney tissue of both genotypes, differences significantly blunted in obstructed kidney tissue of Ampkα1^-/-^ mice as compared to Ampkα1^+/+^ mice (Fig 3C and 3D).

**Fig 4:**
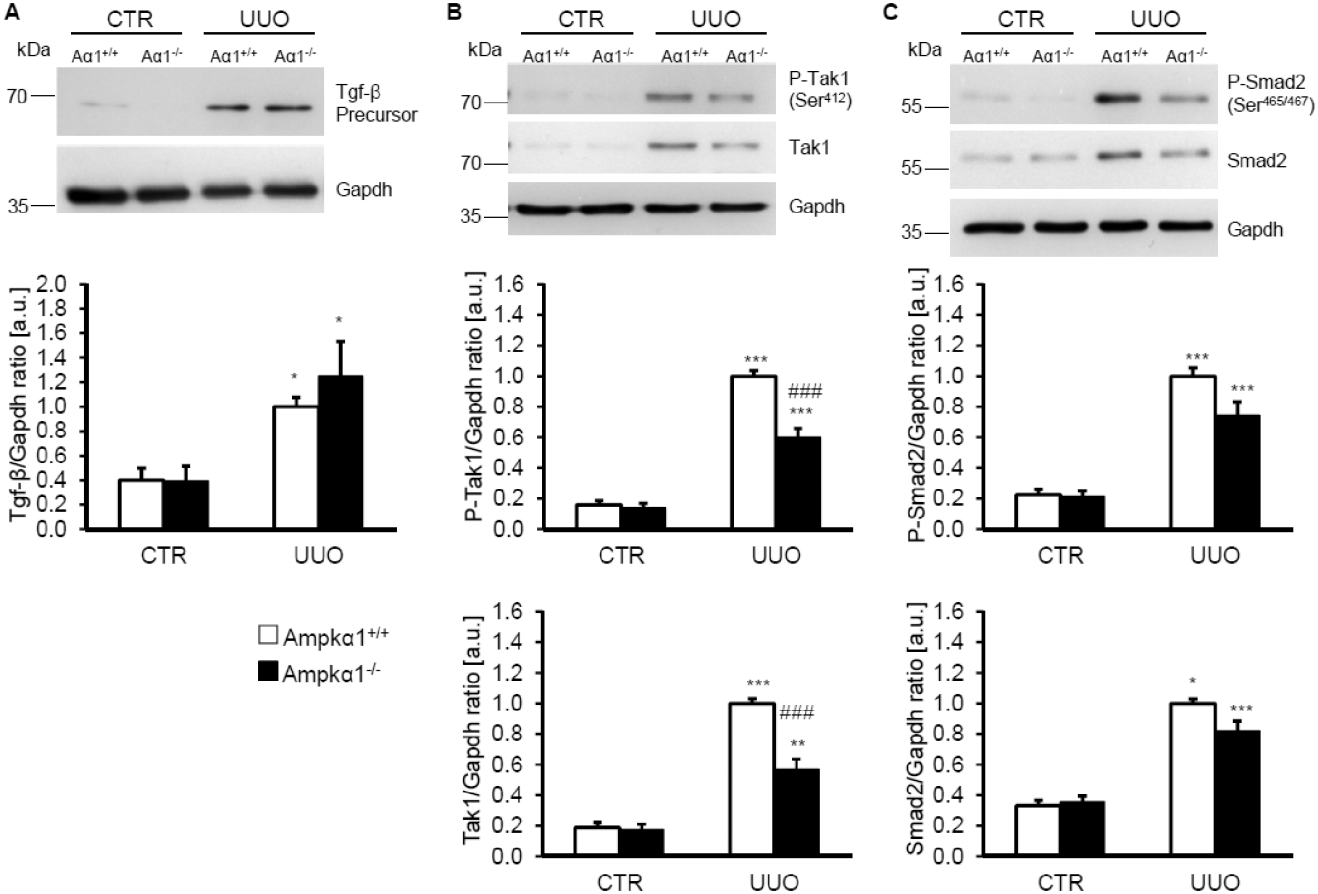
Ampkα1 sensitive up-regulation of Tgf-β-activated protein kinase 1 (Tak1) following unilateral ureteral obstruction. **A.** Representative original Western blots and arithmetic means ± SEM (n = 9) of normalized Tgf-β precursor/Gapdh protein ratio in renal tissue from non-obstructed control kidney (CTR) and obstructed kidney (UUO) of Ampkα1 knockout mice (black bars, Ampkα1^-/-^) and respective wild-type mice (white bars, Ampkα1^+/+^) following 7 days of unilateral ureteral obstruction. **B.** Representative original Western blots and arithmetic means ± SEM (n = 9) of normalized phospho-Tak1 (Ser^412^)/ Gapdh protein ratio and total Tak1/ Gapdh protein ratio in renal tissue from non-obstructed control kidney (CTR) and obstructed kidney (UUO) of Ampkα1 knockout mice (black bars, Ampkα1^-/-^) and respective wild-type mice (white bars, Ampkα1^+/+^) following 7 days of unilateral ureteral obstruction. **C.** Representative original Western blots and arithmetic means ± SEM (n = 9) of normalized phospho-Smad2 (Ser^465/467^)/ Gapdh protein ratio and total Smad2/ Gapdh protein ratio in renal tissue from non-obstructed control kidney (CTR) and obstructed kidney (UUO) of Ampkα1 knockout mice (black bars, Ampkα1^-/-^) and respective wild-type mice (white bars, Ampkα1^+/+^) following 7 days of unilateral ureteral obstruction. *(p<0.05), **(p<0.01), ***(p<0.001) statistically significant vs. control kidney tissues of respective mice; ###(p<0.001) statistically significant vs. respective kidney tissues of wild-type mice.

Further experiments were performed to gain some insight into Ampkα1-dependent signaling following UUO. Following 7 days of obstructive injury, Tgf-β precursor protein abundance was significantly up-regulated in obstructed kidney tissue, an effect not significantly modified by Ampkα1 deficiency (Fig 4A). The protein abundance of phosphorylated and total Tgf-ß-activated kinase (Tak1) were significantly higher in the obstructed kidney tissues than in non-obstructed kidney tissue of either Ampkα1^+/+^ mice or Ampkα1^-/-^ mice, but were significantly lower in the obstructed kidney tissues of Ampkα1^-/-^ mice than in the obstructed kidney tissues of Ampkα1^+/+^ mice (Fig 4B). Furthermore, obstructive injury significantly increased the protein abundance of phosphorylated and total Smad2 in both genotypes, effects not significantly affected by Ampkα1 deficiency (Fig 4C).

**Fig 5:**
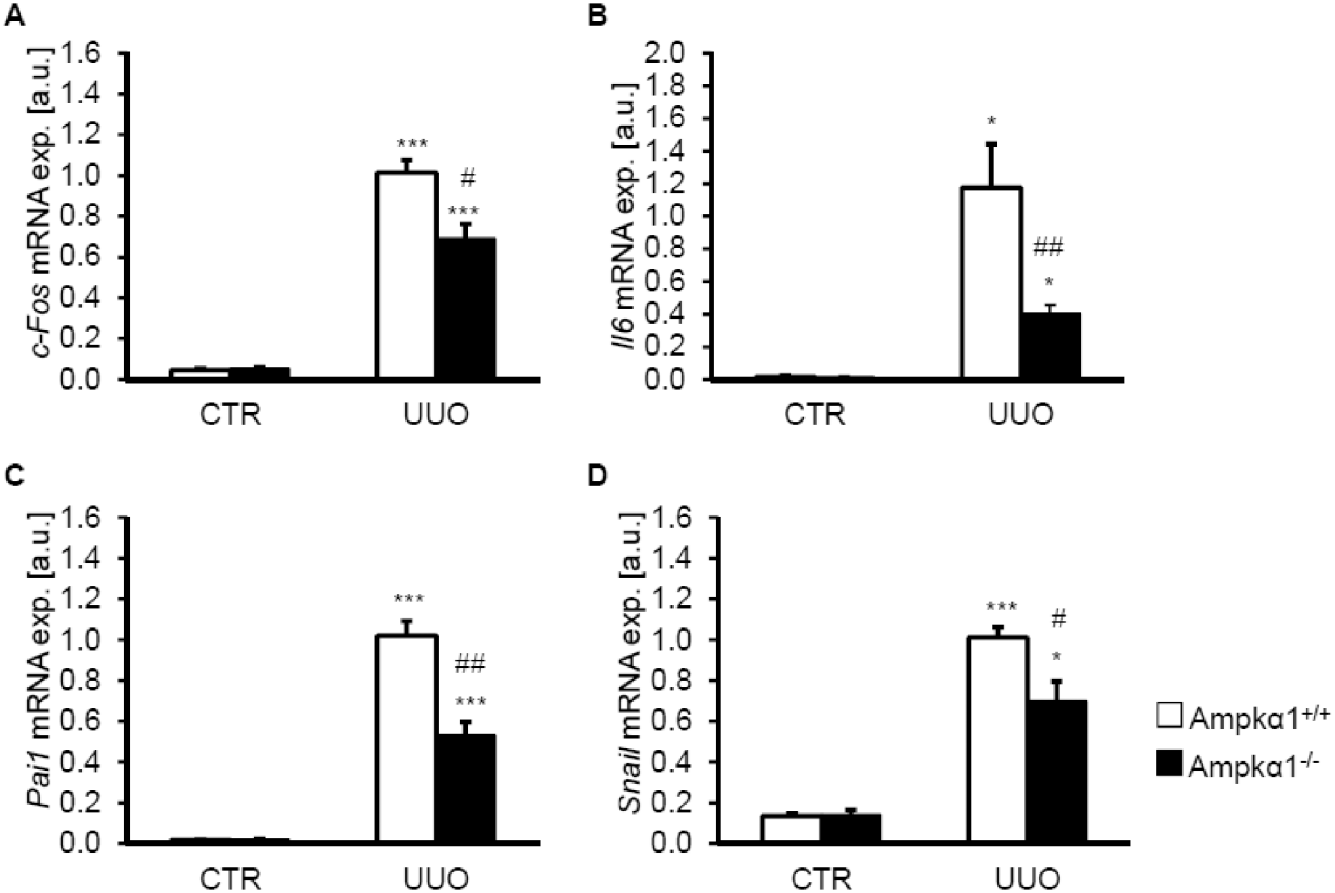
Ampkα1 sensitive up-regulation of Tak1 target genes following unilateral ureteral obstruction. Arithmetic means ± SEM (n = 9) of *c-Fos* (**A**), interleukin 6 (*Il6*, **B**), *Pai1* (**C**) and *Snai1* (**D**) relative mRNA expression in renal tissue from non-obstructed control kidney (CTR) and obstructed kidney (UUO) of Ampkα1 knockout mice (black bars) and respective wild-type mice (white bars) following 7 days of unilateral ureteral obstruction. *(p<0.05), ***(p<0.001) statistically significant vs control kidney tissues of respective mice; #(p<0.05), ##(p<0.01) statistically significant vs. respective kidney tissues of wild-type mice.

To further elucidate Ampkα1-sensitive signaling following UUO, the mRNA levels of Tak1-downstream targets: the transcription factor *c-Fos*, the cytokine interleukin 6 (*Il6*), as well as *plasminogen activator inhibitor 1* (*Pai1*) and the transcription factor *Snai1* were determined by quantitative RT-PCR. As shown in Fig 5A–5D, 7 days of UUO treatment significantly increased the mRNA expression of *c-Fos*, *Il6*, *Pai1* and *Snai1* in the obstructed kidney tissue than in non-obstructed kidney tissue from either Ampkα1^+/+^ mice or Ampkα1^-/-^ mice. The increased mRNA levels of *c-Fos*, *Il6*, *Pai1* and *Snai1* in the obstructed kidney tissue were significantly less pronounced in Ampkα1^-/-^ mice than in Ampkα1^+/+^ mice.

**Fig 6:**
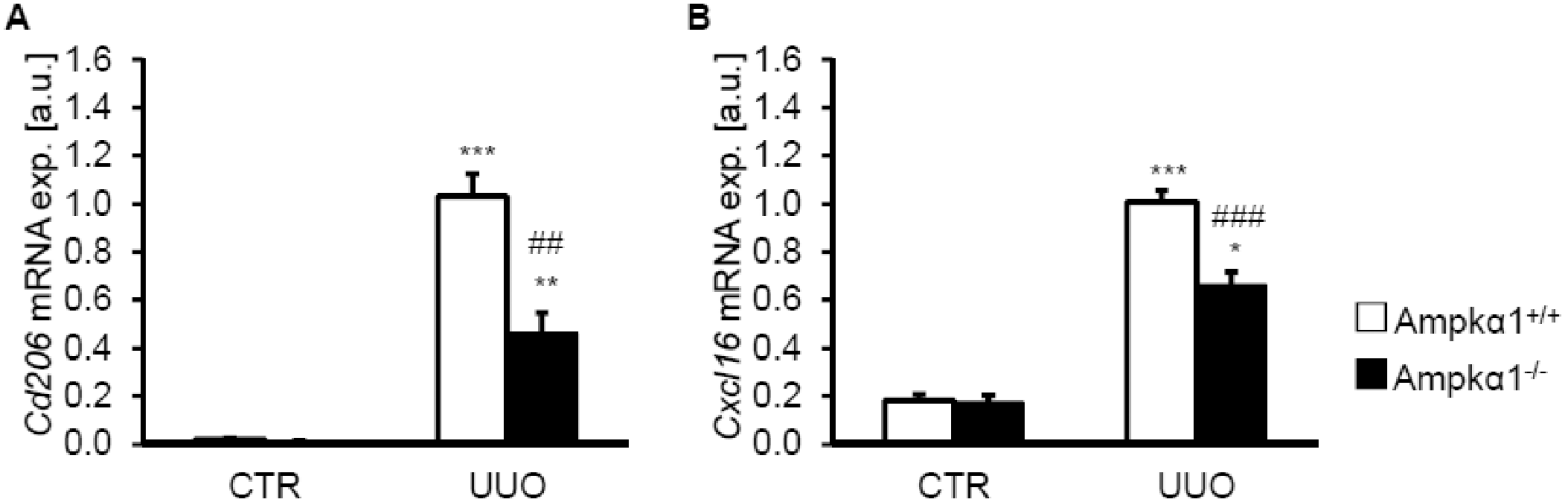
Ampkα1 deficiency attenuates M2 macrophage polarization and pro-fibrotic chemokine production following unilateral ureteral obstruction. Arithmetic means ± SEM (n = 9) of *Cd206* (**A**) and *Cxcl16* (**B**) relative mRNA expression in renal tissue from non-obstructed control kidney (CTR) and obstructed kidney (UUO) of Ampkα1 knockout mice (black bars) and respective wild-type mice (white bars) following 7 days of unilateral ureteral obstruction. *(p<0.05), **(p<0.01), ***(p<0.001) statistically significant vs. control kidney tissues of respective mice; ##(p<0.01), ###(p<0.001) statistically significant vs. respective kidney tissues of wild-type mice.

Next, we explored whether Ampkα1 deficiency affected macrophage polarization and myeloid fibroblast formation following 7 days of obstructive injury. To this end, the mRNA expression of *Cd206*, a marker for M2 macrophages and of *Cxcl16*, a chemokine associated with myeloid fibroblast formation were determined. As shown in Fig 6, the mRNA levels of *Cd206* and *Cxcl16* were significantly higher in the obstructed kidney tissue than in non-obstructed kidney tissue of both genotypes, effects significantly blunted in the obstructed kidney tissue of Ampkα1^-/-^ mice as compared to obstructed kidney tissue of Ampkα1^+/+^ mice.

**Fig 7:**
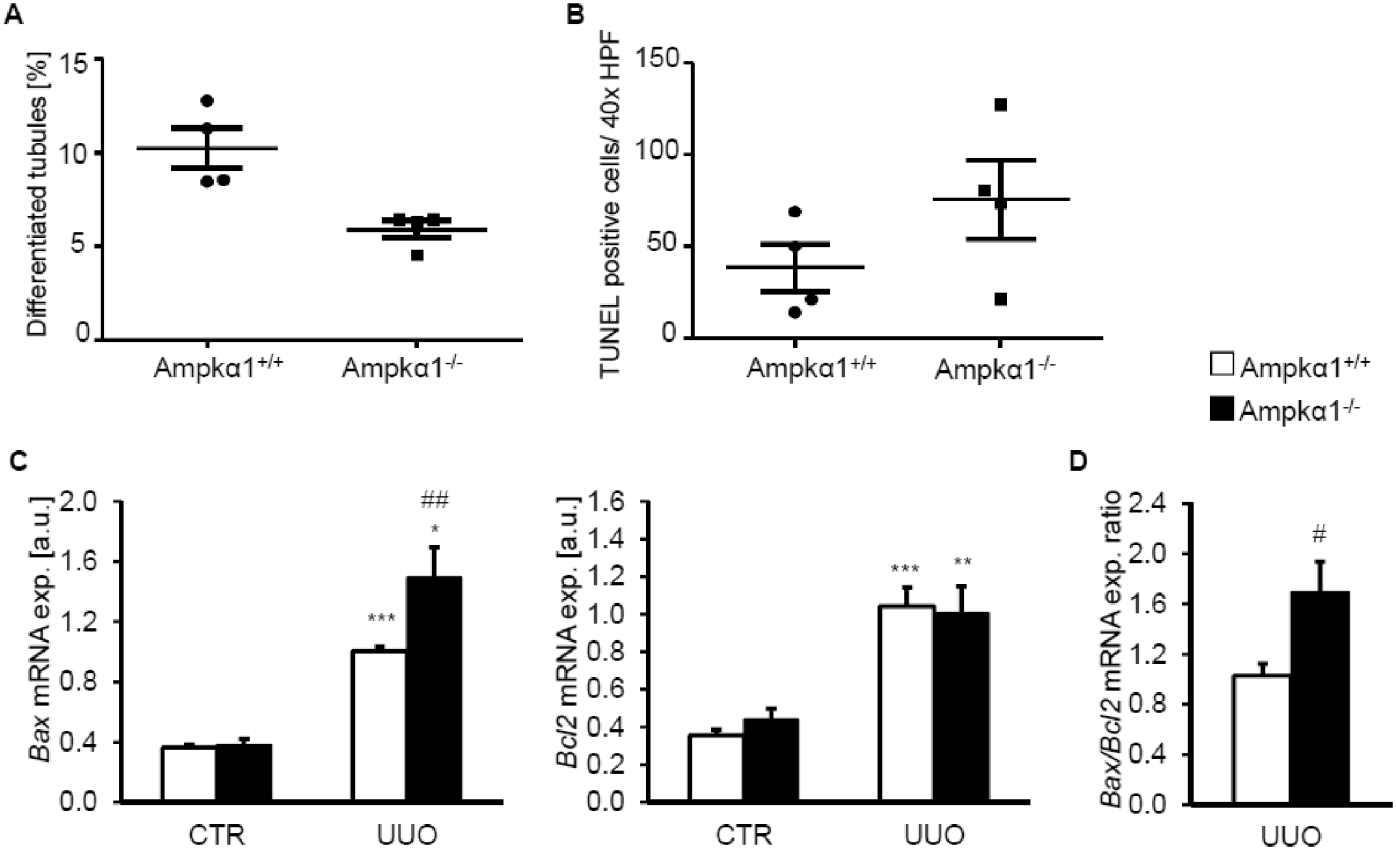
Ampkα1 sensitive renal tubulointerstitial injury and apoptosis following unilateral ureteral obstruction. **A.** Percentage of differentiated tubules in sections of obstructed kidneys from Ampkα1 knockout mice (Ampkα1^-/-^) and respective wild-type mice (Ampkα1^+/+^) following 7 days of unilateral ureteral obstruction. **B.** TUNEL positive cells number/40x HPF in sections of obstructed kidneys from Ampkα1 knockout mice (Ampkα1^-/-^) and respective wild-type mice (Ampkα1^+/+^) following 7 days of unilateral ureteral obstruction. **C.** Arithmetic means ± SEM (n = 9) of *Bax* and *Bcl2* relative mRNA expression in renal tissue from non-obstructed control kidney (CTR) and obstructed kidney (UUO) of Ampkα1 knockout mice (black bars) and respective wild-type mice (white bars) following 7 days of unilateral ureteral obstruction. **D.** Arithmetic means ± SEM (n = 9) of *Bax/Bcl2* relative mRNA expression ratio in renal tissue from obstructed kidney (UUO) of Ampkα1 knockout mice (black bar, Ampkα1^-/-^) and respective wild-type mice (white bar, Ampkα1^+/+^) following 7 days of unilateral ureteral obstruction. *(p<0.05), **(p<0.01), ***(p<0.001) statistically significant vs. control kidney tissues of respective mice; #(p<0.05), ##(p<0.01) statistically significant vs. respective kidney tissues of wild-type mice.

To elucidate the effects of Ampkα1 deficiency on renal function in obstructive nephropathy, renal tubular injury and apoptosis were determined in Ampkα1^+/+^ and Ampkα1^-/-^ mice following 7 days of UUO. As shown in Fig 7A, the percentage of differentiated tubules was higher in the obstructed kidney tissue of Ampkα1^+/+^ mice than in the obstructed kidney tissue of Ampkα1^-/-^ mice. Moreover, the number of TUNEL positive cells tended to be higher in the obstructed kidney tissue of Ampkα1^-/-^ mice than in the obstructed kidney tissue of Ampkα1^+/+^ mice (Fig 7B). Similarly, the ratio of renal *Bax/Bcl2* mRNA levels was significantly increased in the obstructed kidney tissue of Ampkα1^-/-^ mice as compared to Ampkα1^+/+^ mice (Fig 7C and 7D). Collectively, the observations suggest that Ampkα1 deficiency augments apoptosis following obstructive injury.

## Discussion

The present observations suggest that Ampkα1 isoform contributes to myofibroblast formation and renal pro-fibrotic signaling following unilateral ureteral obstruction (UUO). Renal obstruction is followed by up-regulation of Ampkα1 and an isoform shift from Ampkα2 to Ampkα1. The observed isoform shift may result from colonization of renal tissue by myofibroblasts or stimulation of renal Ampkα subunit expression.

The functional significance of Ampkα1 is illustrated by the blunted expression of α-smooth muscle actin (α-Sma) in Ampkα1^-/-^ mice, indicating that Ampkα1 was required for the full effect of unilateral ureteral obstruction on α-Sma expression. It should be kept in mind though, that Ampkα1 may play a permissive role rather than an active role in the up-regulation of α-Sma expression. Moreover, the effect of Ampkα1deficiency on tissue injury following UUO may be indirect, e.g. due to increased abundance of suicidal, phosphatidylserine exposing erythrocytes, anemia and/or splenomegaly [30].

The blunted response of α-Sma expression following UUO in the Ampkα1^-/-^ mice was associated with reduced phosphorylation and expression of Tgf-ß-activated kinase (Tak1). Tak1 is a key factor in the renal fibrotic response [12]. Tak1 inhibition blunts the renal fibrotic response and pro-inflammatory cytokine expression following obstructive injury [42]. Tak1 may activate Ampk and thus amplify Ampk-dependent functions [43, 44]. Conversely, Ampkα1 may activate Tak1, which in turn promotes expression of pro-inflammatory cytokines [14]. Specifically, Tak1 up-regulates the expression of c-Fos [45], Il6 [46], Pai1 [15] and Snai1 [15], the expression of which is important for the effects of obstructive injury [7, 36, 47, 48]. Ampk promotes myofibroblast function and potentiates Tgf-ß-induced α-Sma expression, at least in part, via Tak1 [49].

Il6, a downstream effector of Tak1 [46], promotes M2 macrophage polarization [50]. Cxcl16 plays a crucial role in the accumulation of myeloid fibroblasts during renal fibrosis [51]. Accordingly, up-regulation of the M2 macrophage marker CD206 was blunted in Ampkα1-deficient obstructed kidney tissues. M2 macrophages cause increased α-Sma expression in the kidney [52].

UUO further up-regulates adiponectin, which activates Ampk following obstructive kidney injury [53]. Ampk is involved in the signaling of adiponectin-induced myofibroblast accumulation [53]. Adiponectin deficiency is associated with a reduced fibrotic response following obstructive injury, involving expression of CD206 and Cxcl16 induced by adiponectin [53]. The pro-fibrotic effects of adiponectin require Ampk [53]. Adiponectin is further increased in patients suffering from end-stage renal disease [54]. The pro-fibrotic effects of adiponectin-Ampk may thus contribute to increased pro-fibrotic signaling in end-stage renal disease [2, 53]. Thus, up-regulation of Tak1 presumably contributes to Ampkα1 sensitive pro-fibrotic signaling following UUO. Ampkα1 may promote M2 macrophage infiltration via Tak1-induced Il6 expression, but also other factors might influence Ampk-dependent effects following obstructive injury.

However, Ampkα1 deficiency did not exert a profound effect on collagen deposition. This finding is in accordance with previous observations: Ampkα1 deficiency blunts the α-Sma expression following myocardial infarction [55]. However, collagen expression was not profoundly altered by Ampkα1 deficiency in myocardial infarction and a specific effect on myofibroblast activation and α-Sma expression was observed [55] Impaired M2 macrophage activation similarly leads to impaired myofibroblast activation in myocardial infarction [56]. Activation of Ampk promotes the angiotensin II-induced proliferation of cardiac fibroblasts [57].

Conflicting observations have been reported on the effects of the Ampk activator, 5-aminoimidazole-4-carboxyamide ribonucleoside (AICAR). At the one hand, AICAR has been shown to blunt the fibrotic response following obstructive injury [34]. Similarly, metformin attenuates the epithelial-to-mesenchymal transition *in vitro* [33]. In contrast, following myocardial infarction, AICAR promotes scar formation and myofibroblast activation [58]. The effects of AICAR and metformin are, however, not necessarily due to activation of Ampk, as both exert Ampk-independent effects [59]. Beyond that, the two Ampkα isoforms do not necessarily regulate identical targets and the Ampkα1 isoform may exert effects distinct from those of Ampkα2 [19]. Accordingly, Ampkα2, but not Ampkα1 deficiency exacerbates cardiac remodeling [60]. In failing hearts, a shift from Ampkα1 to Ampkα2 was observed [61]. The Ampkα2 isoform seems to block myofibroblast trans-differentiation [35]. Furthermore, Ampkα2 is a suppressor of endothelial inflammation [62]. On the other hand, constitutively active Ampkα1 expression in endothelial cells causes vascular inflammation [63]. The observed isoform shift of Ampkα2 towards Ampkα1 in obstructed kidney tissues may therefore contribute to the pro-fibrotic signaling events.

The reduced α-Sma expression following obstructive injury was associated with an increased tubular dilation and Bax/Bcl2 ratio. Inhibition of Ampkα1 was previously shown to sensitize cells for apoptosis [14].

As a first line of defence against energy depletion Ampkα1 attempts to restore cellular energy by triggering a variety of mechanisms including stimulation of cellular glucose uptake by up-regulation of the facilitative glucose carriers GLUT1, GLUT2, GLUT3, GLUT4 and the Na^+^-coupled glucose transporter SGLT1 as well as stimulation of glycolysis, fatty acid oxidation and expression of several enzymes required for ATP production [22, 27, 28]. All those functions counteract ATP depletion. The enhanced apoptosis and tissue injury in Ampkα1^-/-^ mice following UUO indeed suggest that cell survival following UUO is compromised by Ampk deficiency. If the effects of Ampk on ATP production and consumption fail to restore cellular energy, the survival of the energy consuming cells is jeopardized and cells may undergo cell death. The partial replacement of energy consuming cells in an ischemic tissue by energy sparing fibrous tissue supports the survival of the residual cells, which compete for the delivered oxygen and fuel. Thus, stimulation of tissue fibrosis by myofibroblast activation may be a useful fundamental mechanism overcoming an imbalance between delivery and consumption of energy in a given tissue.

In conclusion, UUO leads to an isoform shift towards Ampkα1 in renal tissue. Ampkα1 is up-regulated following obstructive nephropathy and participates in the orchestration of mechanisms conferring cell survival and fostering tissue fibrosis.

## Supporting Information

S1 File Renal Acc phosphorylation following unilateral ureteral obstruction in wild-type and Ampkα1 knockout mice. Representative original Western blots and arithmetic means ± SEM (n = 9) of normalized phospho-Acc (Ser^79^)/ Gapdh and total Acc/Gapdh protein ratio in renal tissue from non-obstructed control kidney (CTR) and obstructed kidney (UUO) of Ampkα1 knockout mice (black bars, Ampkα1^-/-^) and respective wild-type mice (white bars, Ampkα1^+/+^) following 7 days of unilateral ureteral obstruction.
(TIF)

S2 File Renal Ampkα isoform expression following 3 days of unilateral ureteral obstruction. **A.** Representative original Western blots showing Ampkα1 and Gapdh protein expression in renal tissue from non-obstructed control kidney (CTR) and obstructed kidney (UUO) of Ampkα1 knockout mice (Aα1^-/-^) and respective wild-type mice (Aα1^+/+^) following 3 days of unilateral ureteral obstruction. Arithmetic means ± SEM (n = 8) of normalized Ampkα1/Gapdh protein ratio in renal tissue from non-obstructed control kidney (CTR) and obstructed kidney (UUO) of wild-type mice (Ampkα1^+/+^) following 3 days of unilateral ureteral obstruction (UUO). *(p<0.05) statistically significant vs. control kidney tissues of wild-type mice. **B.** Representative original Western blots and arithmetic means ± SEM (n = 8) of normalized Ampkα2/Gapdh protein ratio in renal tissue from non-obstructed control kidney (CTR) and obstructed kidney (UUO) of Ampkα1 knockout mice (black bars, Ampkα1^-/-^) and respective wild-type mice (white bars, Ampkα1^+/+^) following 3 days of unilateral ureteral obstruction. **C.** Representative original Western blots and arithmetic means ± SEM (n = 8) of normalized phospho-Ampkα (Thr^172^)/Gapdh and total Ampkα/Gapdh protein ratio in renal tissue from non-obstructed control kidney (CTR) and obstructed kidney (UUO) of Ampkα1 knockout mice (black bars, Ampkα1^-/-^) and respective wild-type mice (white bars, Ampkα1^+/+^) following 3 days of unilateral ureteral obstruction. **(p<0.01), ***(p<0.001) statistically significant vs. control kidney tissues of respective mice; #(p<0.05), ##(p<0.01), ###(p<0.001) statistically significant vs. respective kidney tissues of wild-type mice.
(TIF)

S3 File α-Smooth muscle actin expression following 3 days of unilateral ureteral obstruction in Ampkα1 deficiency. Representative original Western blots and arithmetic means ± SEM (n = 8) of normalized α-smooth muscle actin (α-Sma)/Gapdh protein ratio in renal tissue from non-obstructed control kidney (CTR) and obstructed kidney (UUO) of Ampkα1 knockout mice (black bars, Ampkα1^-/-^) and respective wild-type mice (white bars, Ampkα1^+/+^) following 3 days of unilateral ureteral obstruction. **(p<0.01), ***(p<0.001) statistically significant vs. control kidney tissues of respective mice.
(TIF)

S4 File Renal Ampkα isoform expression following 3 weeks of unilateral ureteral obstruction. **A.** Representative original Western blots showing Ampkα1 and Gapdh protein expression in renal tissue from non-obstructed control kidney (CTR) and obstructed kidney (UUO) of Ampkα1 knockout mice (Aα1^-/-^) and respective wild-type mice (Aα1^+/+^) following 3 weeks of unilateral ureteral obstruction. Arithmetic means ± SEM (n = 7) of normalized Ampkα1/Gapdh protein ratio in renal tissue from non-obstructed control kidney (CTR) and obstructed kidney (UUO) of wild-type mice (Ampkα1^+/+^) following 3 weeks of unilateral ureteral obstruction (UUO). *(p<0.05) statistically significant vs. control kidney tissues of wild-type mice. **B.** Representative original Western blots and arithmetic means ± SEM (n = 7) of normalized Ampkα2/Gapdh protein ratio in renal tissue from non-obstructed control kidney (CTR) and obstructed kidney (UUO) of Ampkα1 knockout mice (black bars, Ampkα1^-/-^) and respective wild-type mice (white bars, Ampkα1^+/+^) following 3 weeks of unilateral ureteral obstruction. **C.** Representative original Western blots and arithmetic means ± SEM (n = 7) of normalized phospho-Ampkα (Thr^172^)/Gapdh and total Ampkα/Gapdh protein ratio in renal tissue from non-obstructed control kidney (CTR) and obstructed kidney (UUO) of Ampkα1 knockout mice (black bars, Ampkα1^-/-^) and respective wild-type mice (white bars, Ampkα1^+/+^) following 3 weeks of unilateral ureteral obstruction. *(p<0.05), **(p<0.01), ***(p<0.001) statistically significant vs. control kidney tissues of respective mice; ###(p<0.001) statistically significant vs. respective kidney tissues of wild-type mice.
(TIF)

S5 File Ampkα1 sensitive α-smooth muscle actin expression following 3 weeks of unilateral ureteral obstruction. Representative original Western blots and arithmetic means ± SEM (n = 7) of normalized α-smooth muscle actin (α-Sma)/Gapdh protein ratio in renal tissue from non-obstructed control kidney (CTR) and obstructed kidney (UUO) of Ampkα1 knockout mice (black bars, Ampkα1^-/-^) and respective wild-type mice (white bars, Ampkα1^+/+^) following 3 weeks of unilateral ureteral obstruction. ***(p<0.001) statistically significant vs. control kidney tissues of respective mice. ###(p<0.001) statistically significant vs. respective kidney tissues of wild-type mice.
(TIF)
