# Supplementary figures and images for: Impact of AMP-Activated Protein Kinase α1 Deficiency on Tissue Injury following Unilateral Ureteral Obstruction
Source: PLoS One. 2015 Aug 18;10(8):e0135235. doi: 10.1371/journal.pone.0135235 (PMC4540418; doi:10.1371/journal.pone.0135235)

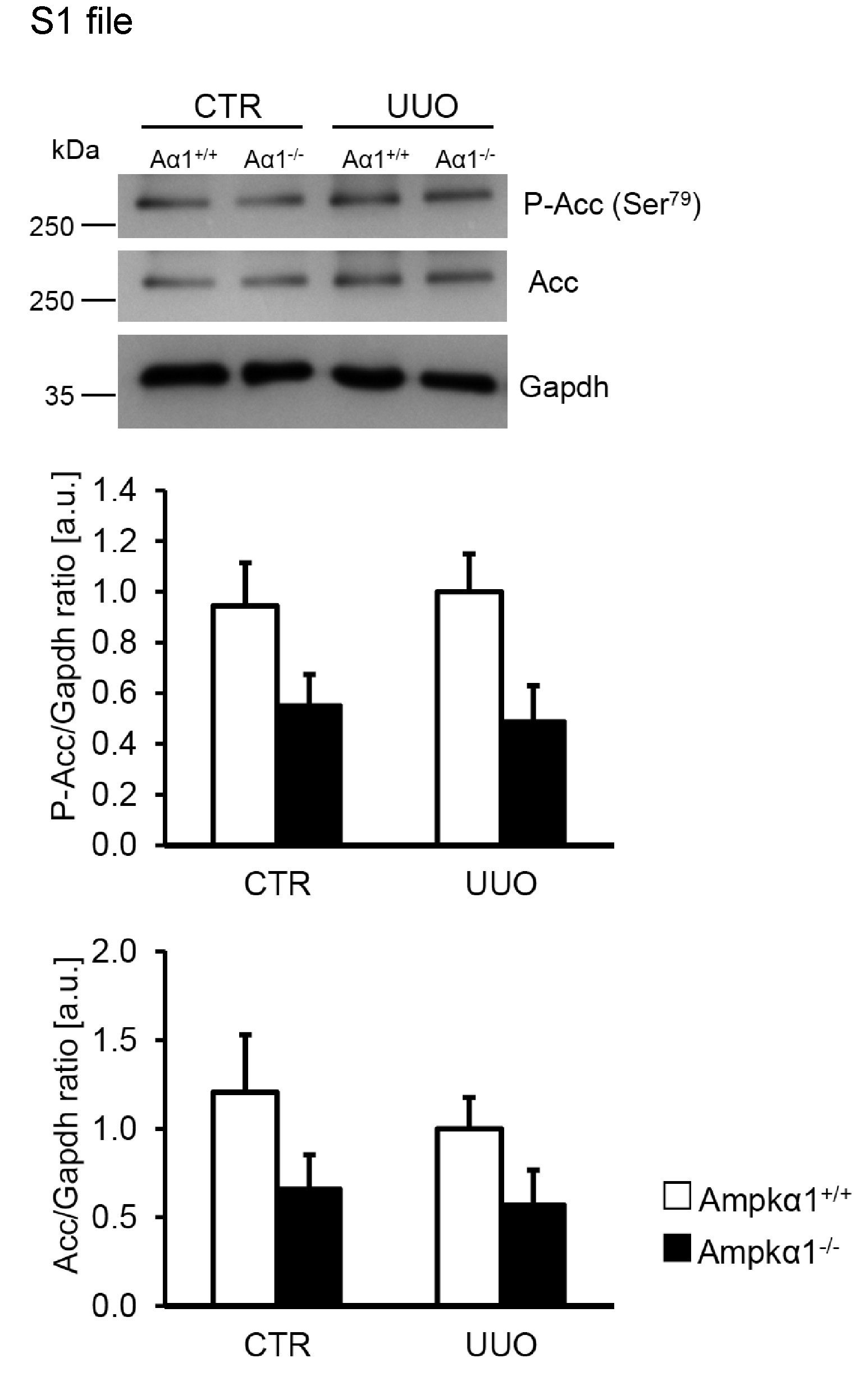

Supplement: S1 File — Representative original Western blots and arithmetic means ± SEM (n = 9) of normalized phospho-Acc (Ser79)/ Gapdh and total Acc/Gapdh protein ratio in renal tissue from non-obstructed control kidney (CTR) and obstructed kidney (UUO) of Ampkα1 knockout mice (black bars, Ampkα1-/-) and respective wild-type mice (white bars, Ampkα1+/+) following 7 days of unilateral ureteral obstruction. (TIF) [file pone.0135235.s001.tif]

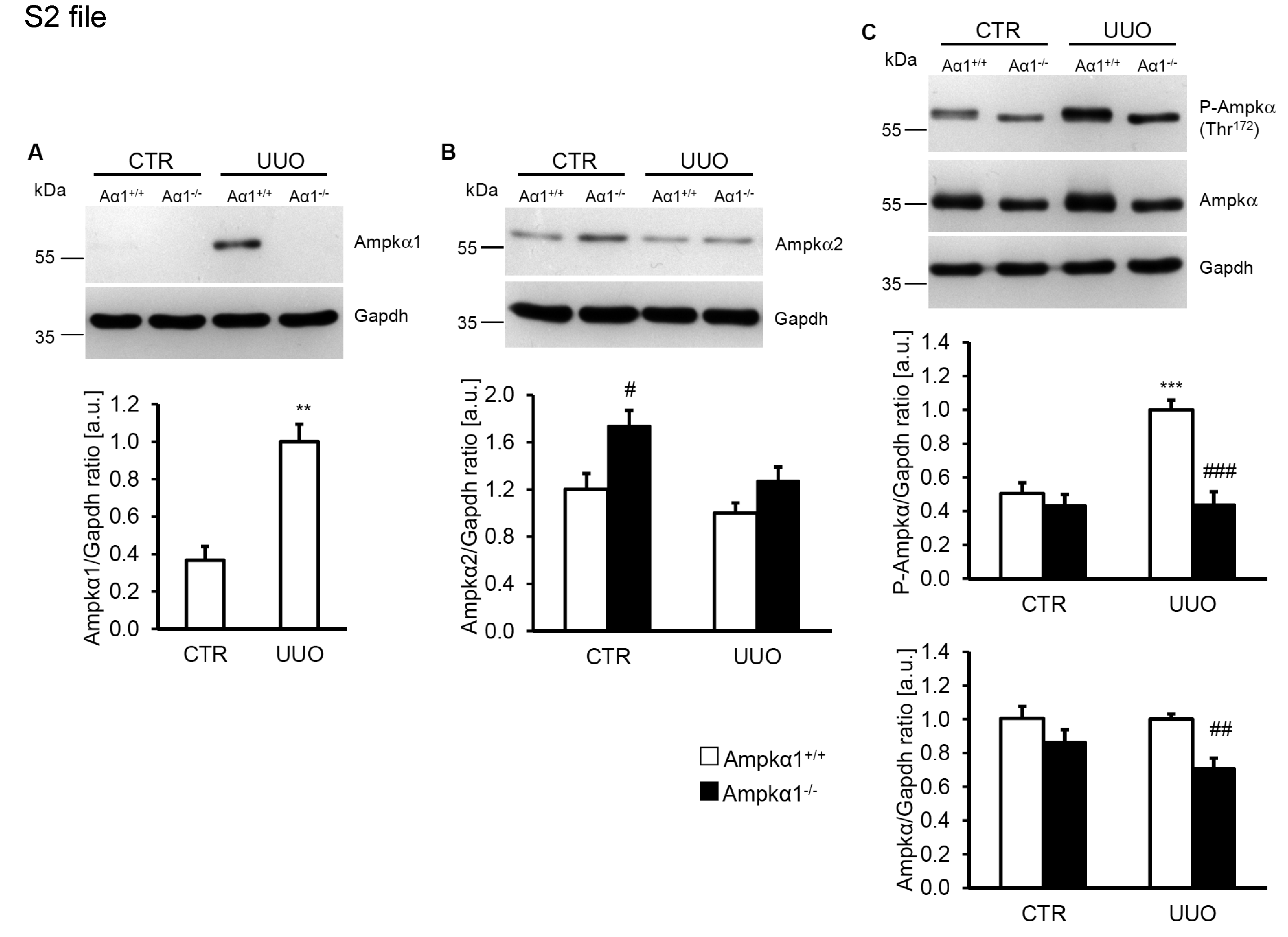

Supplement: S2 File — A. Representative original Western blots showing Ampkα1 and Gapdh protein expression in renal tissue from non-obstructed control kidney (CTR) and obstructed kidney (UUO) of Ampkα1 knockout mice (Aα1-/-) and respective wild-type mice (Aα1+/+) following 3 days of unilateral ureteral obstruction. Arithmetic means ± SEM (n = 8) of normalized Ampkα1/Gapdh protein ratio in renal tissue from non-obstructed control kidney (CTR) and obstructed kidney (UUO) of wild-type mice (Ampkα1+/+) following 3 days of unilateral ureteral obstruction (UUO). *(p<0.05) statistically significant vs. control kidney tissues of wild-type mice. B. Representative original Western blots and arithmetic means ± SEM (n = 8) of normalized Ampkα2/Gapdh protein ratio in renal tissue from non-obstructed control kidney (CTR) and obstructed kidney (UUO) of Ampkα1 knockout mice (black bars, Ampkα1-/-) and respective wild-type mice (white bars, Ampkα1+/+) following 3 days of unilateral ureteral obstruction. C. Representative original Western blots and arithmetic means ± SEM (n = 8) of normalized phospho-Ampkα (Thr172)/Gapdh and total Ampkα/Gapdh protein ratio in renal tissue from non-obstructed control kidney (CTR) and obstructed kidney (UUO) of Ampkα1 knockout mice (black bars, Ampkα1-/-) and respective wild-type mice (white bars, Ampkα1+/+) following 3 days of unilateral ureteral obstruction. **(p<0.01), ***(p<0.001) statistically significant vs. control kidney tissues of respective mice; #(p<0.05), ##(p<0.01), ###(p<0.001) statistically significant vs. respective kidney tissues of wild-type mice. (TIF) [file pone.0135235.s002.tif]

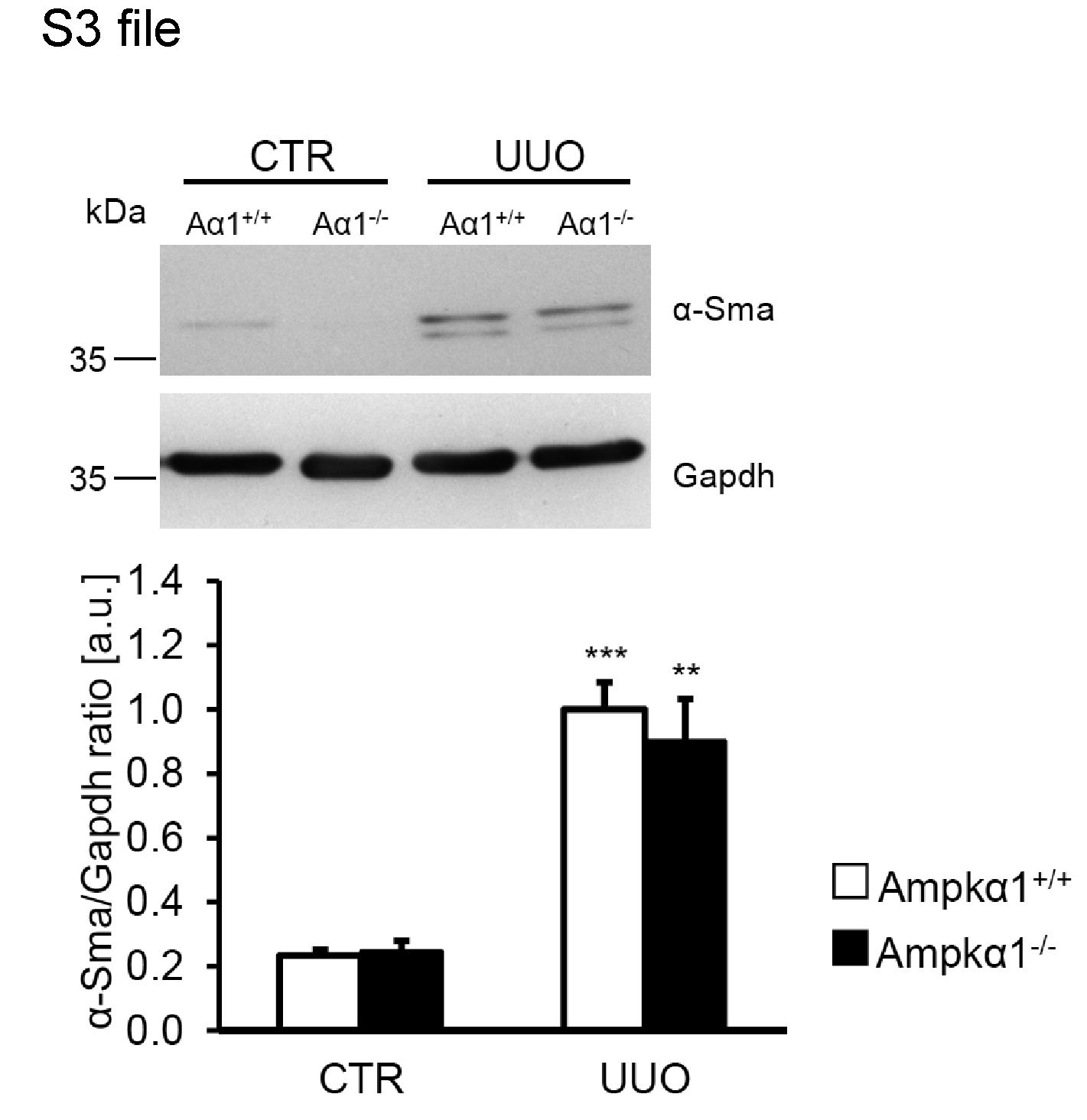

Supplement: S3 File — Representative original Western blots and arithmetic means ± SEM (n = 8) of normalized α-smooth muscle actin (α-Sma)/Gapdh protein ratio in renal tissue from non-obstructed control kidney (CTR) and obstructed kidney (UUO) of Ampkα1 knockout mice (black bars, Ampkα1-/-) and respective wild-type mice (white bars, Ampkα1+/+) following 3 days of unilateral ureteral obstruction. **(p<0.01), ***(p<0.001) statistically significant vs. control kidney tissues of respective mice. (TIF) [file pone.0135235.s003.tif]

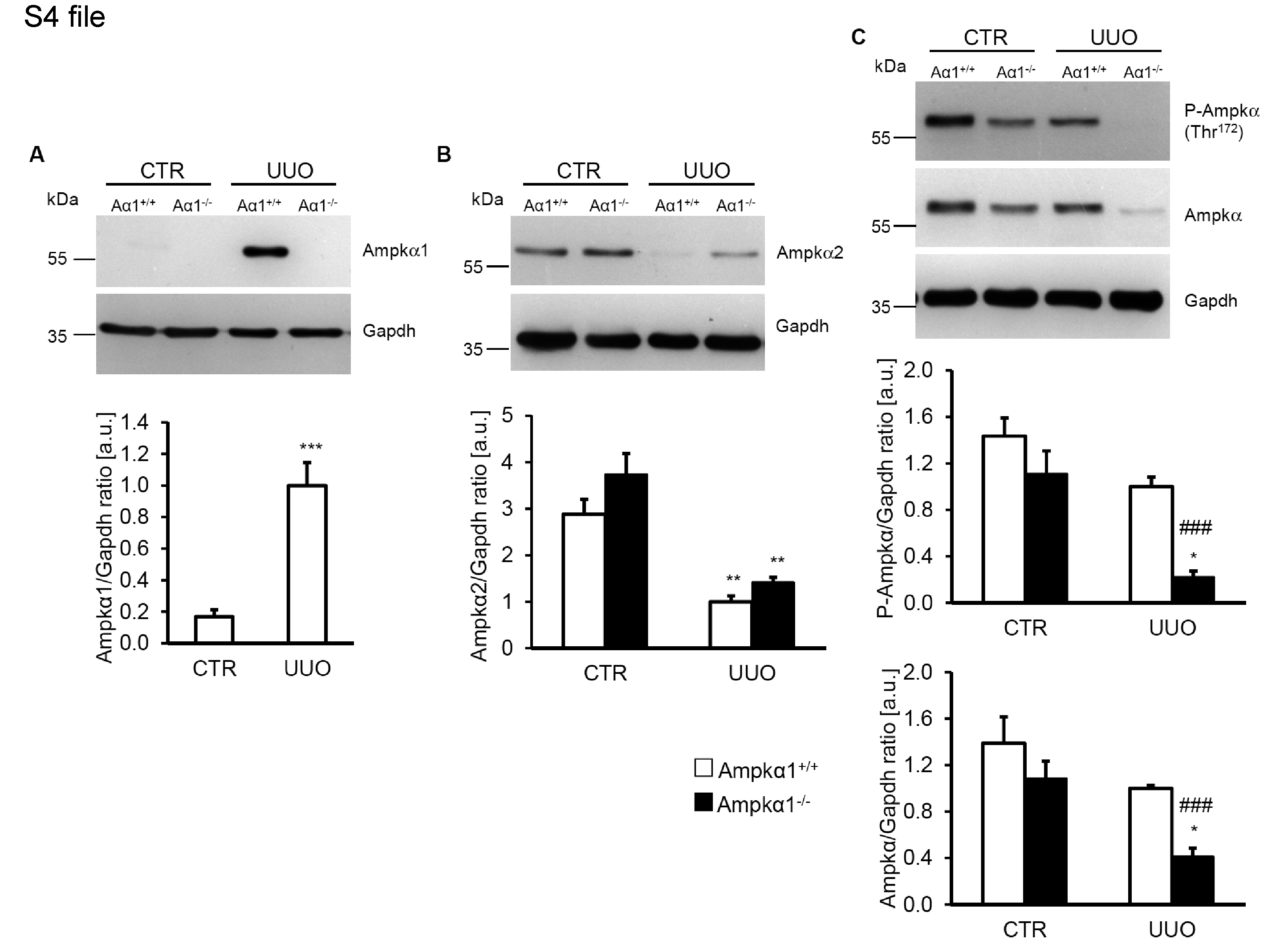

Supplement: S4 File — A. Representative original Western blots showing Ampkα1 and Gapdh protein expression in renal tissue from non-obstructed control kidney (CTR) and obstructed kidney (UUO) of Ampkα1 knockout mice (Aα1-/-) and respective wild-type mice (Aα1+/+) following 3 weeks of unilateral ureteral obstruction. Arithmetic means ± SEM (n = 7) of normalized Ampkα1/Gapdh protein ratio in renal tissue from non-obstructed control kidney (CTR) and obstructed kidney (UUO) of wild-type mice (Ampkα1+/+) following 3 weeks of unilateral ureteral obstruction (UUO). *(p<0.05) statistically significant vs. control kidney tissues of wild-type mice. B. Representative original Western blots and arithmetic means ± SEM (n = 7) of normalized Ampkα2/Gapdh protein ratio in renal tissue from non-obstructed control kidney (CTR) and obstructed kidney (UUO) of Ampkα1 knockout mice (black bars, Ampkα1-/-) and respective wild-type mice (white bars, Ampkα1+/+) following 3 weeks of unilateral ureteral obstruction. C. Representative original Western blots and arithmetic means ± SEM (n = 7) of normalized phospho-Ampkα (Thr172)/Gapdh and total Ampkα/Gapdh protein ratio in renal tissue from non-obstructed control kidney (CTR) and obstructed kidney (UUO) of Ampkα1 knockout mice (black bars, Ampkα1-/-) and respective wild-type mice (white bars, Ampkα1+/+) following 3 weeks of unilateral ureteral obstruction. *(p<0.05), **(p<0.01), ***(p<0.001) statistically significant vs. control kidney tissues of respective mice; ###(p<0.001) statistically significant vs. respective kidney tissues of wild-type mice. (TIF) [file pone.0135235.s004.tif]

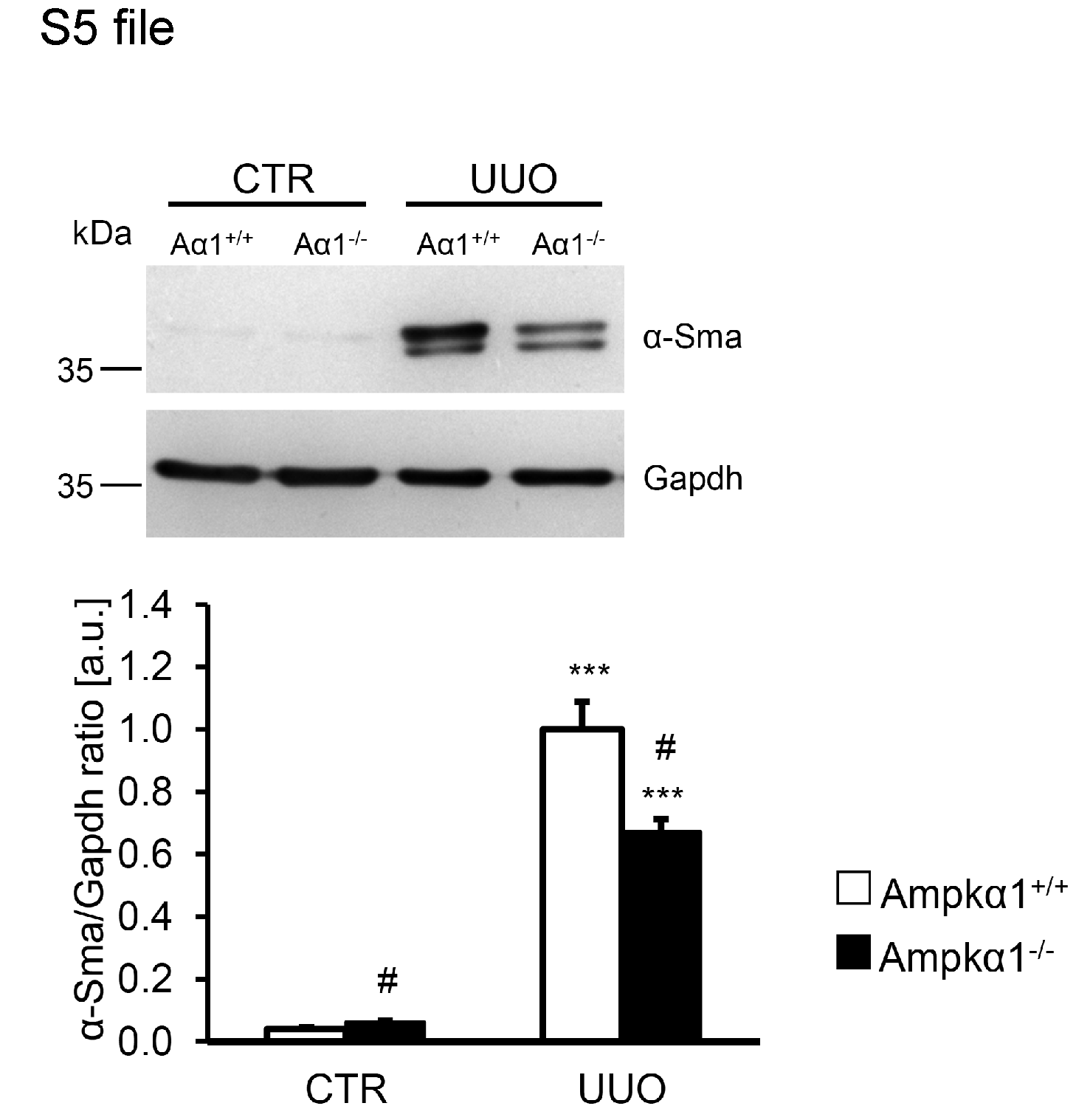

Supplement: S5 File — Representative original Western blots and arithmetic means ± SEM (n = 7) of normalized α-smooth muscle actin (α-Sma)/Gapdh protein ratio in renal tissue from non-obstructed control kidney (CTR) and obstructed kidney (UUO) of Ampkα1 knockout mice (black bars, Ampkα1-/-) and respective wild-type mice (white bars, Ampkα1+/+) following 3 weeks of unilateral ureteral obstruction. ***(p<0.001) statistically significant vs. control kidney tissues of respective mice. ###(p<0.001) statistically significant vs. respective kidney tissues of wild-type mice. (TIF) [file pone.0135235.s005.tif]
